# Supplementary material for: Bone marrow involvement in patients with metastatic castration sensitive prostate cancer
Source: PLoS One. 2022 Jul 21;17(7):e0270956. doi: 10.1371/journal.pone.0270956 (PMC9302741; doi:10.1371/journal.pone.0270956)
Supplement: Attachment — Submitted filename: BM rebuttal PLOS 3 18.docx [file pone.0270956.s002.docx]

Dr. Bauckneht

Managing editor

PLOS ONE

RE: “Bone Marrow Involvement in Patients with Metastatic Castration Sensitive Prostate Cancer”

(Manuscript ID PONE-D-21-40910)

Dear Dr. Bauckneht:

Thank you for considering our manuscript for possible publication. We appreciate the comments of the reviewers and we have revised the manuscript accordingly.

Hereby the revised version of the :  “Bone Marrow Involvement in Patients with Metastatic Castration Sensitive Prostate Cancer” (Manuscript ID PONE-D-21-40910)

We have listed the concerns of the reviewers and have detailed our actions to address these issues, point by point.

To summarize the most important changes, we have:

-We updated the methodology section to include the ethical approval.

-We added a statement regarding the data availability.

Finally, we want to thank you and the reviewers, for the effort to review this manuscript.

I hope we were able to address all reviewers’ concerns satisfactorily.

Looking forward to hearing back soon from you.

Sincerely,

Samer Salah

1. Please ensure that your manuscript meets PLOS ONE's style requirements, including those for file naming. The PLOS ONE style templates can be found at

https://journals.plos.org/plosone/s/file?id=wjVg/PLOSOne_formatting_sample_main_body.pdf and

<https://journals.plos.org/plosone/s/file?id=ba62/PLOSOne_formatting_sample_title_authors_affiliations.pdf>

2. Please provide additional details regarding participant consent. In the ethics statement in the Methods and online submission information, please ensure that you have specified (1) whether consent was informed and (2) what type you obtained (for instance, written or verbal, and if verbal, how it was documented and witnessed). If your study included minors, state whether you obtained consent from parents or guardians. If the need for consent was waived by the ethics committee, please include this information.

If you are reporting a retrospective study of medical records or archived samples, please ensure that you have discussed whether all data were fully anonymized before you accessed them and/or whether the IRB or ethics committee waived the requirement for informed consent. If patients provided informed written consent to have data from their medical records used in research, please include this information.

3. Thank you for stating the following financial disclosure:

Response:

“No Funding”

At this time, please address the following queries:

a)        Please clarify the sources of funding (financial or material support) for your study. List the grants or organizations that supported your study, including funding received from your institution.

Response:

None

b)        State what role the funders took in the study. If the funders had no role in your study, please state: “The funders had no role in study design, data collection and analysis, decision to publish, or preparation of the manuscript.”

Response:

None

c)        If any authors received a salary from any of your funders, please state which authors and which funders.

Response:

None

d)        If you did not receive any funding for this study, please state: “The authors received no specific funding for this work.”

Response:

The statement was added “The authors received no specific funding for this work.”

Please include your amended statements within your cover letter; we will change the online submission form on your behalf.

4. In your Data Availability statement, you have not specified where the minimal data set underlying the results described in your manuscript can be found. PLOS defines a study's minimal data set as the underlying data used to reach the conclusions drawn in the manuscript and any additional data required to replicate the reported study findings in their entirety. All PLOS journals require that the minimal data set be made fully available. For more information about our data policy, please see http://journals.plos.org/plosone/s/data-availability.

Upon re-submitting your revised manuscript, please upload your study’s minimal underlying data set as either Supporting Information files or to a stable, public repository and include the relevant URLs, DOIs, or accession numbers within your revised cover letter. For a list of acceptable repositories, please see http://journals.plos.org/plosone/s/data-availability#loc-recommended-repositories. Any potentially identifying patient information must be fully anonymized.

Important: If there are ethical or legal restrictions to sharing your data publicly, please explain these restrictions in detail. Please see our guidelines for more information on what we consider unacceptable restrictions to publicly sharing data: http://journals.plos.org/plosone/s/data-availability#loc-unacceptable-data-access-restrictions. Note that it is not acceptable for the authors to be the sole named individuals responsible for ensuring data access.

We will update your Data Availability statement to reflect the information you provide in your cover letter.

Response:

We totally believe in the importance of data availability. However, the IRB committee at our institution did not grant us the right to publicly sharing the data as there are no legislation in our country to regulate sharing such data publicly; and this might put the authors and the institution at high legal liability in our country. However, the data might be available if requested , by contacting the IRB office at King Hussein Cancer Center to grant us approval of data sharing.  Contacting the IRB will commence once you request the data.

5. PLOS requires an ORCID iD for the corresponding author in Editorial Manager on papers submitted after December 6th, 2016. Please ensure that you have an ORCID iD and that it is validated in Editorial Manager. To do this, go to ‘Update my Information’ (in the upper left-hand corner of the main menu), and click on the Fetch/Validate link next to the ORCID field. This will take you to the ORCID site and allow you to create a new iD or authenticate a pre-existing iD in Editorial Manager. Please see the following video for instructions on linking an ORCID iD to your Editorial Manager account: <https://www.youtube.com/watch?v=_xcclfuvtxQ>

Response:

Added Samer Salah : 0000-0001-7421-2522

6. Your ethics statement should only appear in the Methods section of your manuscript. If your ethics statement is written in any section besides the Methods, please move it to the Methods section and delete it from any other section. Please ensure that your ethics statement is included in your manuscript, as the ethics statement entered into the online submission form will not be published alongside your manuscript.

Response:

Moved to the method section

7. Please review your reference list to ensure that it is complete and correct. If you have cited papers that have been retracted, please include the rationale for doing so in the manuscript text, or remove these references and replace them with relevant current references. Any changes to the reference list should be mentioned in the rebuttal letter that accompanies your revised manuscript. If you need to cite a retracted article, indicate the article’s retracted status in the References list and also include a citation and full reference for the retraction notice.

Response:

The references were double checked and complies with the journal style.

[Note: HTML markup is below. Please do not edit.]

Reviewers' comments:

Reviewer's Responses to Questions

**Comments to the Author**

1. Is the manuscript technically sound, and do the data support the conclusions?

The manuscript must describe a technically sound piece of scientific research with data that supports the conclusions. Experiments must have been conducted rigorously, with appropriate controls, replication, and sample sizes. The conclusions must be drawn appropriately based on the data presented.

Reviewer #1: Yes

Reviewer #2: Yes

2. Has the statistical analysis been performed appropriately and rigorously?

Reviewer #1: Yes

Reviewer #2: Yes

3. Have the authors made all data underlying the findings in their manuscript fully available?

The [PLOS Data policy](http://www.plosone.org/static/policies.action#sharing) requires authors to make all data underlying the findings described in their manuscript fully available without restriction, with rare exception (please refer to the Data Availability Statement in the manuscript PDF file). The data should be provided as part of the manuscript or its supporting information, or deposited to a public repository. For example, in addition to summary statistics, the data points behind means, medians and variance measures should be available. If there are restrictions on publicly sharing data—e.g. participant privacy or use of data from a third party—those must be specified.

Reviewer #1: Yes

Reviewer #2: Yes

4. Is the manuscript presented in an intelligible fashion and written in standard English?

PLOS ONE does not copyedit accepted manuscripts, so the language in submitted articles must be clear, correct, and unambiguous. Any typographical or grammatical errors should be corrected at revision, so please note any specific errors here.

Reviewer #1: Yes

Reviewer #2: Yes

5. Review Comments to the Author

Please use the space provided to explain your answers to the questions above. You may also include additional comments for the author, including concerns about dual publication, research ethics, or publication ethics. (Please upload your review as an attachment if it exceeds 20,000 characters)

Reviewer #1: Very interesting idea, specially in the setting of metastatic castrate sensitive prostate cancer. Different approaches are being developed to subselect patients at higher risk of progression before progressing to crpc state.

It was not clear in the abstract and the full manuscript if all the 189 patients subject of the study, underwent a bone marrow biopsy. I am assuming it is the case. But I think it should be mentioned.

Response:

Thank you for your comment. Actually, BM bx is not a standard of care in our institution and we don’t perform BM on all these patients. It was a subset of patients who had BM bx in other institution for unknown reasons and referred for treatment at ours.

We added this statement to the methodology section *:” Only eleven patients (6%) had bone marrow biopsy  in other institutions and were referred to our institution for prostate cancer management.”*

Also we highlighted this in our limitation section :*Finally, all of these patients were referred to our institution with bone morrow biopsy/aspirate was done in other institutions; therefore, we were not able to control for selection bias and other factors affected the decision of the referring physician to perform the bone marrow biopsy.*

Reviewer #2: The article is a retrospective review of clinical data that aims to determine the clinical importance of bone marrow metastasis of prostate cancer on the patient prognosis and survival. It is an important topic, the analysis includes a relevant number of patients and is based on observation of images clinical and laboratory data.
The article is well written, with a good flow and easy to read for a non-expert audience, but also with sufficient technical depth.

The statistical analysis is appropriate, and the conclusions correspond to the clinical observations. The authors acknowledge their limitations accordingly, with scientific soundness.

The novelty of the article is moderate but adds relevant information to the field that sums to the overall knowledge of prostate cancer.
The absence of typos and grammar issues also reveals that the authors performed an appropriate and extensive proof reading,

The article deserves to be published and this reviewer have one minor edit in the article:

The article would increase its relevance including images of the bone metastasis of prostate cancer that were used in the diagnosis MRI or CT scans.

Response:

Thank you for your comments. BM metastasis was based on BM biopsy aspirate not images.
